# Chinese Color Nest Project : An accelerated longitudinal brain-mind cohort

**DOI:** 10.1016/j.dcn.2021.101020

**Published:** 2021-10-11

**Authors:** Siman Liu, Yin-Shan Wang, Qing Zhang, Quan Zhou, Li-Zhi Cao, Chao Jiang, Zhe Zhang, Ning Yang, Qi Dong, Xi-Nian Zuo, The Chinese Color Nest Consortium

**Affiliations:** a Research Center for Lifespan Development of Mind and Brain, Institute of Psychology, Chinese Academy of Sciences, Beijing 100101, China; b Department of Psychology, University of Chinese Academy of Science, Beijing 100049, China; c State Key Laboratory of Cognitive Neuroscience and Learning, Beijing Normal University, Beijing 100875, China; d Developmental Population Neuroiscience Research Center, International Data Group/McGovern Institute for Brain Research, Beijing Normal University, Beijing 100875, China; e School of Psychology, Capital Normal University, Beijing 100048, China; f Department of Psychology, College of Education, Hebei Normal University, Shijiazhuang 05024, Hebei, China

**Keywords:** Adolescence, School-age children, Brain growth curve, Accelerated longitudinal design, Lifespan development, Data sharing

## Abstract

The ongoing Chinese Color Nest Project (CCNP) was established to create normative charts for brain structure and function across the human lifespan, and link age-related changes in brain imaging measures to psychological assessments of behavior, cognition, and emotion using an accelerated longitudinal design. In the initial stage, CCNP aims to recruit 1520 healthy individuals (6–90 years), which comprises three phases: developing (devCCNP: 6–18 years, *N* = 480), maturing (matCCNP: 20–60 years, *N* = 560) and aging (ageCCNP: 60–84 years, *N* = 480). In this paper, we present an overview of the devCCNP, including study design, participants, data collection and preliminary findings. The devCCNP has acquired data with three repeated measurements from 2013 to 2017 in Southwest University, Chongqing, China (CCNP-SWU, *N* = 201). It has been accumulating baseline data since July 2018 and the second wave data since September 2020 in Chinese Academy of Sciences, Beijing, China (CCNP-CAS, *N* = 168). Each participant in devCCNP was followed up for 2.5 years at 1.25-year intervals. The devCCNP obtained longitudinal neuroimaging, biophysical, social, behavioral and cognitive data via MRI, parent- and self-reported questionnaires, behavioral assessments, and computer tasks. Additionally, data were collected on children’s learning, daily life and emotional states during the COVID-19 pandemic in 2020. We address data harmonization across the two sites and demonstrated its promise of characterizing the growth curves for the overall brain morphometry using multi-center longitudinal data. CCNP data will be shared via the National Science Data Bank and requests for further information on collaboration and data sharing are encouraged.

## 1 Introduction

Construction of normative charts for brain structure and function across the human lifespan in typically developing individuals is essential for characterizing brain morphology and connectivity changes and monitoring neurodevelopmental status (Harms et al., 2018, Peterson et al., 2018). The age-related trajectory of the brain in the life cycle can be used to elucidate the normative pattern of brain development, maturation and aging, and further reveal similarities and differences at different developmental stages (Betzel et al., 2014, Courchesne et al., 2000, Zuo et al., 2010, Zuo et al., 2017). Growth charts for height, weight and head circumference provide important information about children’s physical health, which enables pediatricians and parents to identify abnormal physical growth (Cole, 2012, Kløvgaard et al., 2018, Ouyang et al., 2018, Yang et al., 2015). Similarly, inadequate or excessive brain growth as estimated by deviations from the normalized distribution in growth curves may represent abnormal neurodevelopmental status that needs closer monitoring or interventions (Peterson et al., 2018).

With respect to the use of brain growth charts in clinical practices, many neuropsychiatric disorders, such as autism spectrum disorder (ASD), psychotic disorder, bipolar disorder and attention deficit hyperactivity disorder (ADHD), are theorized to have origins in abnormal brain development (Ecker et al., 2015, Hazlett et al., 2017, Jalbrzikowski et al., 2019, Norman et al., 2016, Skåtun et al., 2016). The presence and characterization of deviations from normative brain trajectories related to neuropsychiatric conditions may help reveal associated pathological and physiological changes, allowing for the construction of neuroimaging biomarkers and shedding new light on clinical diagnosis and treatment (Marquand et al., 2019, Shaw, 2016, Zabihi et al., 2019, Ziegler et al., 2014). From an intervention perspective, brain growth charts also provide optimal periods for intervention and normative age-expected values that are required for further assessment of the effectiveness of an intervention program (Kulkarni et al., 2017). If participants with abnormal brain development return to follow the normative pattern after receiving a specific intervention, this intervention could be considered effective in attenuating neurocognitive impairment or promoting brain development and successful aging.

Given the implications of brain growth charts in basic research and clinical application, researchers have sought to establish brain growth charts and use them to predict cognitive deficits and neuropsychiatric conditions (Erus et al., 2015, Kessler et al., 2016). However, reliable and accurate charts for the brain are still not available (see a recent seminal work from Bethlehem et al., 2021). The underlying reason is that most studies rely on cross-sectional design and focus on a narrow range of ages and sample sizes, which limit their ability to accurately reflect the continuity of individual development over the life course. In cross-sectional studies, the age-related changes shown in developmental trajectories often confound age and cohort effects (Spuling et al., 2015, Sutin et al., 2013, Twenge et al., 2017). Standard (i.e., pure or single-cohort) longitudinal design overcomes the shortcomings of cross-sectional design by recruiting a single cohort of participants of the same age and following them over a period of time. However, this design inevitably raises issues of time demands and sample attrition over time, and also elicits practice effects due to repeated assessments, which reduces its feasibility and efficiency in life-span studies involving a wide range of age groups (Thompson et al., 2011, Hayward and Krause, 2016, Twenge et al., 2017, Zuo et al., 2017).

The dilemma between cross-sectional and standard longitudinal designs highlights the need for using accelerated longitudinal designs (ALD), which recruit multiple cohorts of individuals of different ages and follow them over a shorter period of time (Galbraith et al., 2017, Joiner et al., 2018). The multicohort approach is less time-consuming and more practical for creating growth curves over a long interval of the life course. Indeed, previous studies have mapped developmental trajectories over a time span of decades using ALD, the most famous of which is The Seattle Longitudinal Study, investigating longitudinally cognitive development from young adulthood to old age (Schaie et al., 2004, Hülür et al., 2019). In the field of developmental cognitive neuroscience, researchers have used brain imaging methods to create accelerated longitudinal cohorts (see Table 1 for a non-exhaustive list). These studies have characterized the developmental trajectories of brain function and cognitive processes from childhood to adulthood, and further examine the neural correlates underlying the developmental changes of cognitive performances (Darki and Klingberg, 2015, Montez et al., 2017). For example, Simmonds et al. (2017) conducted an accelerated longitudinal study over 10 years in 129 healthy participants, aged 8–30 years, who were asked to return for annual visits (mean = 2.8 visits/participant), and found that increases in encoding/retrieval activity in visual cortex accompanied increases in working memory accuracy while decreases in maintenance activity in prefrontal/subcortical regions accompanied decreases in working memory latency. Despite these advances, there are very few accelerated longitudinal cohorts (e.g., LCBC) available for characterizing brain and psycho-behavioral development across the postnatal lifespan. Meanwhile, previous studies revealed cultural or ethnic effects on development of brain and mind (Fan et al., 2015, Dong et al., 2020; Qiu, 2020). Many large-scale neuroimaging samples include almost universally European or American (see Fig. 1 in Rosenberg et al., 2018), missing cultural diversity.

**Table 1:** A non-exhaustive list of normative developmental samples obtained by accelerated longitudinal design using multimodal MRI methods.

Sample or Lab	Age Range (baseline)	Waves (visits)	Interval (months)	Country Region	Study Resource Website or/and Key Recent Reference (s)
NCANDA	12–21	4	~12	USA	http://ncanda.org/overview.php (Meruelo et al., 2021, Silveira et al., 2021)
LunaCog	9–26	2–6	~12	USA	https://lncd.pitt.edu/wp (Parr et al., 2021, Larsen et al., 2020, Calabro et al., 2020)
NCDL	4–8	3	~12	USA	http://ncdl.umd.edu/index.html (Canada et al., 2021, Canada et al., 2020, Riggins and Spencer, 2020)
ISLA	6–45	2–5	18–176	USA	Prigge et al., 2021;Lange et al., 2015;Lainhart, 2015
Enhanced NKI	6–18	3	15	USA	http://fcon_1000.projects.nitrc.org/indi/enhanced (Nooner et al., 2012)
Americleft	7–27	2–4	24–72	USA	https://www.americleft.org (Kuhlmann et al., 2021, Crerand et al., 2017)
NIH-PD/CPB	5–25	2–6	~24	USA	https://pediatricmri.nih.gov (Russell et al., 2021, Giedd et al., 2015, Evans, 2006)
TottenhamDAN	4–16	2–3	~24	USA	https://danlab.psychology.columbia.edu (VanTieghem et al., 2021)
BrainTime	8–29	3	~24	Netherlands	http://www.juniorhersenen.nl/braintime (van Duijvenvoorde et al., 2019)
YOUth	8–16	3	~36	Netherlands	https://www.uu.nl/en/research/youth-cohort-study (Onland-Moret et al., 2020)
KaessGroup	9–16	3	~12	Germany	https://www.upd.unibe.ch/research (Mürner-Lavanchy et al., 2020)
LEAP	6–30	2	12–24	EU	https://www.eu-aims.eu/the-leap-study (Zabihi et al., 2020, Loth et al., 2017)
c-VEDA	6–23	3	12	UK, India	https://kcl.ac.uk/cveda (Zhang et al., 2020, Holla et al., 2020a, Holla et al., 2020)
CBD	6–12	2+	~12	China	Hao et al., 2021, Fan et al., 2021, Schumann et al., 2019, Tao, 2019
NESDA	18–65	3	~24	Netherlands	https://www.nesda.nl/nesda-english (Grasby et al., 2020, Penninx et al., 2008)
Betula	25–100	6	~60	Sweden	https://umu.se/en/research/projects/betula---aging-memory-and-dementia
PREVENT-AD	55–88	5	~6	Canada	https://openpreventad.loris.ca (Tremblay-Mercier et al., 2021)
VLS	53–95	3	~48	Canada	https://sites.ualberta.ca/~vlslab (Peters et al., 2020, Hertzog et al., 2019)
BABRI	5+	2+	24–36	China	http://babri.bnu.edu.cn (Yang et al., 2021, Chen et al., 2021, Gao et al., 2019)
Cam-CAN	18–88	3	12–24	UK	https://cam-can.org (Bethlehem et al., 2020, Shafto et al., 2014)
LCBC	4–90+	3	2.5–80	Norway	https://www.oslobrains.no (Fjell et al., 2021, Fjell et al., 2015, Walhovd et al., 2016)
CCNP	4–85	3	15/27	China	http://deepneuro.bnu.edu.cn/?p = 163 (Dong et al., 2021, Dong et al., 2020, Zuo et al., 2017)

**Fig. 1:**
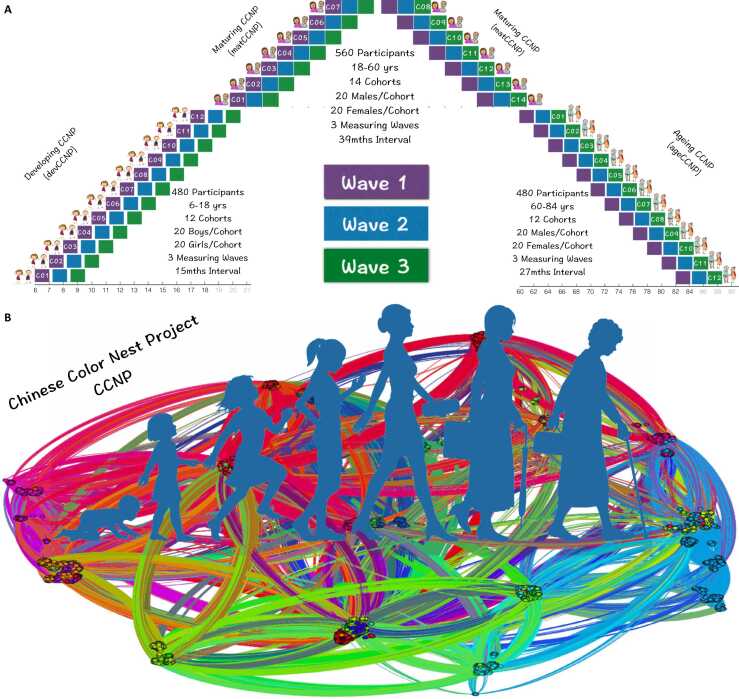
Accelerated longitudinal design and naming for CCNP. (A) CCNP design comprises three phases: developing (devCCNP: 6–18 years, *N*=480, 12 age cohorts, interval = 15 months), maturing (matCCNP: 18–60 years, *N*=560, 14 age cohorts, interval = 39 months) and aging (ageCCNP: 60–84 years, *N*=480, 12 age cohorts, interval = 27 months). It has three waves of measurements including baseline at Wave 1 (purple), followup1 at Wave 2 (blue) and followup2 at Wave 3 (green). There are 40 participants (20 males and 20 females) in each age cohort. (B) A high-resolution network map of the human brain connectome, which was shaped like a colored nest, and inspired us to name the project.

The principal work of the ongoing Chinese Color Nest Project (CCNP) is to collect high-quality data on brain magnetic resonance imaging (MRI) scans and socio-psycho-behavioral factors using an ALD encompassing three repeated measurements on healthy individuals aged 6–84 years at enrollment. The overall aim of CCNP is to build normative charts for brain structure and function across the human lifespan, and to further examine how age-related changes in brain anatomical and function are related to those in psycho-behavioral functions, such as cognitive and behavioral abilities. As illustrated in Fig. 1A, the general CCNP design comprises three phases: developing (devCCNP: 6–18 years, *N* = 24 n), maturing (matCCNP: 18–60 years, *N* = 28 n) and aging (ageCCNP: 60–84 years, *N* = 24 n) and n is the number of sex-specific participants (female or male) in each age cohort. At its pilot stage, CCNP targets an accelerated longitudinal cohort of 1520 healthy participants (i.e., n = 20) implemented at two cities (Chongqing and Beijing). Specifically, the devCCNP started in 2013, which has successfully acquired CCNP-Southwest University (CCNP-SWU) samples with three repeated measurements at the trial stage (2013–2017). At the second stage, devCCNP is collecting CCNP-Chinese Academy of Sciences (CCNP-CAS) baseline data since July 2018 and the second-wave data since September 2020. Meanwhile, the matCCNP was initiated in 2015 and has collected more than 1000 repeated measures in Beijing. This dataset will be published soon as part of an open resource called 3R-BRAIN, for reliable, reproducible and replicable brain imaging research (Zuo et al., 2014, Zuo et al., 2017, Zuo et al., 2019). The ageCCNP will be initiated in 2022 at Beijing and Chongqing, respectively. In this paper, we present an overview of devCCNP, including the study design, participants, data collection and preliminary findings.

## 2 Materials and methods

### 2.1 Naming

Before we started this project, we were finishing a high-resolution (4 mm) network map of the human brain connectome using functional imaging data from more than 1000 healthy volunteers across different stages of the postnatal lifespan (6–85 years) based upon the 1000 Functional Connectomes Project (Biswal et al., 2010, Zuo et al., 2012). This map was shaped like a colored nest and inspired us to name the project (Fig. 1B). In addition, color nest represents protection, vitality and development, and implies the concept of respecting individual differences of human beings and promoting healthy development and well-being. CCNP is devoted to collecting data on brain structure and function across different stages of the human lifespan. While CCNP was currently at its pilot stage and designed to cover the postnatal development (6–90 years), we plan to expand the CCNP design to include participants much younger in future, and even fetuses.

### 2.2 Study design and participants

The CCNP uses the accelerated longitudinal design (ALD), which allows controlling for cohort and time of measurement effects with the additional advantage of collecting data on a wider age range in less time (Thompson et al., 2011, Rioux and Little, 2020). This structured multi-cohort longitudinal design is particularly advantageous for lifespan trajectory studies, and optimal when accounting for missing data. As illustrated in Fig. 1A, at the pilot stage, CCNP distributes the ALD (baseline age = 6–18 years, 12 age cohorts, 3 waves, interval = 15 months) to devCCNP, and the ALD (baseline age = 60–84 years, 12 age cohorts, 3 waves, interval = 27 months) to ageALD while matALD receives the ALD (baseline age = 18–60 years, 14 age cohorts, 3 waves, interval = 39 months). While attrition is an important factor for us to decide the specific sampling intervals, we also consider other scientific factors for developmental brain-mind association studies. The CCNP design ensures an overlap of two measurements between successive age cohorts. The specific sampling intervals indicate that the duration between two neighboring waves is 1.25 years, 2.25 years or 3.25 years, i.e., integral years and one season, preventing multiple visits from the same participant during a season (Spring, Summer, Fall, Winter). As a result of the season effect balanced, for a certain participant, three repeated measurement occasions (i.e., waves) were distributed in various seasons. We provided additional details on the ALD design and participants below.

The trial stage implementation of devCCNP took place at Beibei District, Chongqing, China from March 2013 to January 2017. The Institute of Psychology, Chinese Academy of Sciences (CAS) and the Faculty of Psychology, Southwest University (SWU) are responsible for this pilot study called CCNP-SWU. The ongoing CCNP-CAS began in September 2017 in Beijing, China. Ethical approval was provided by the Institutional Review Board of the CAS Institute of Psychology. To provide better representation of the Chinese population, we aimed to include participants from cities differing in economy and region of the country. CCNP-SWU recruited participants in Chongqing, a second-tier city located in the southwest of China. By contrast, CCNP-CAS recruited participants in Beijing, a first-tier city located in the north of China. Going forward, CCNP will recruit additional participants from more cities (48 cities in next ten years). Furthermore, to ensure demographic consistency across the three phases of CCNP (i.e., devCCNP, matCCNP and ageCCNP), each sub-sample from one of the three phases (e.g., devCCNP) would be recruited to match to other sub-sample from different phases (e.g., matCCNP or age CCNP) across a broad range of social and demographic features. We noted that the measures in the devCCNP would be compared to those that are being used in the matCCNP and ageCCNP if the measures are used for all age groups (e.g., biophysical measures, and MRI scans), or a set of tests designed and normed for different age groups, such as Eysenck Personality Questionnaire and Wechsler Intelligence Scale.

**Fig. 2:**
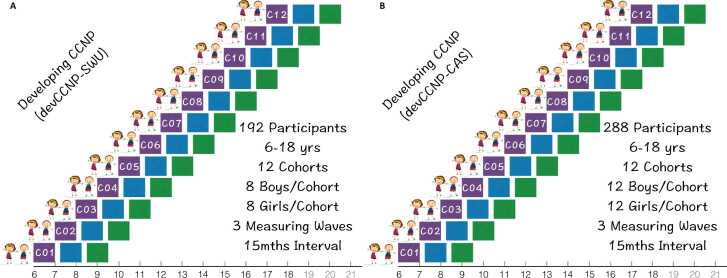
Accelerated longitudinal implementations for devCCNP-SWU and devCCNP-CAS. (A) devCCNP-SWU aimed to recruit 192 participants (8 boys and 8 girls in each cohort, 12 age cohorts). (B) devCCNP-CAS contains 288 participants (12 boys and 12 girls in each cohort, 12 age cohorts). Three measuring waves are represented by purple (Wave 1), blue (Wave 2) and green (Wave 3) boxes. The duration between two neighboring waves is designed as 1.25 years for the purpose of avoiding a kid always visited at a same season. Each cohort covers one integer age-interval at its baseline (C01: 6–7 years; C02: 7–8 years; …; C12: 17–18 years).

The pilot devCCNP aimed to recruit 480 age-sex stratified children distributed in 12 age cohorts at Chongqing (Fig. 2A, CCNP-SWU: 8 boys and 8 girls in each cohort) and Beijing (Fig. 2B, CCNP-CAS: 12 boys and 12 girls in each cohort). A priori power analysis conducted in G*Power 3.1 (Faul et al., 2009) suggested that a sample size of 123 was necessary to achieve the specified statistical power of 0.95 (two tails, a medium effect size f2 = 0.15, alpha = 0.01, and three predictors) for a single regression coefficient in linear multiple regression. It is challenging to estimate sample sizes for building growth curves, because it is potentially more complicate and thus need much larger sample than a common regression. During the pilot stage, the consortium aims to obtain 4560 samples from 1520 participants, which is about 37 times of the common regression derived sample size.

### 2.3 Non-imaging measures

The measures used in devCCNP across all waves to assess biophysical health and socio-psycho-behavioral factors are documented in Table 2. Objective biophysical measures were collected at each wave, including height, weight, head circumference, and biomarkers of cardiovascular health (i.e., blood pressure and pulse). Regarding psychological measures, the study primarily focused on cognition, personality, and issues pertaining to social-emotional functioning. Widely used instruments with high reliability and validity were selected. The questionnaires were primarily built to collect socio-psycho-behavioral data on sociodemographic characteristics, child behavioral problems (Achenbach and Edelbrock, 1983), life events (Liu et al., 1991), self-concept (Piers, 1984), emotions and affects such as stress, anxiety, depression, loneliness, and positive and negative affect (Asher et al., 1984, Cohen et al., 1983, Kovacs, 1992, La Greca et al., 1988, March et al., 1997, Spielberger, 1983, Watson et al., 1988), emotional quotient (Bar-On and Parker, 2000), reading (Xue et al., 2013), personality characteristics (Eysenck and Eysenck, 1975), creativity (Hwang et al., 2007, Torrance, 1984), as well as video game use. Questionnaires on sociodemographic information and CBCL were filled out by the parents, while the rest of the questionnaires were completed by the children.

**Table 2:** List of measures used in devCCNP across all waves.

	Applicable Age Range	Wave 1	Wave 2	Wave 3
**Biophysical Measures**				
Height, Weight, Head Circumference	–	√	√	√
Cardiovascular (Blood pressure, Pulse)	–	√	√	√
**Parent Questionnaires**				
Sociodemographic Questionnaire	–	√		
Child Behavior CheckList (CBCL)	6–16	√	√	√
**Child Questionnaires**				
Adolescent Self-Rating Life Events Checklist (ASLEC)	13–20	√	√	√
Perceived Stress Scale (PSS)	> 10	√	√	√
Piers-Harris Children’s Self-concept Scale (PHCSS)	6–17	√		
Social Anxiety Scale for Children (SASC)	7–16	√	√	√
Multidimensional Anxiety Scale for Children (MASC)	8–19	√	√	√
State-Trait Anxiety Inventor (STAI)	> 6	√	√	√
Children’s Depression Inventory (CDI)	7–17	√	√	√
Children’s Loneliness Scale (CLS)	6–12	√	√	√
Positive Affect and Negative Affect Scale (PANAS)	> 6	√	√	√
BarOn Emotional Quotient Inventory Youth Version	7–18	√	√	√
Eysenck Personality Questionnaire (EPQ, Children’s Version)	7–15	√	√	√
Torrance Test of Creative Thinking (TTCT)	–	√		
Williams Creative Test	–	√	√	√
Literacy Tests (Character Naming and Reading Fluency)	5–12	√	√	√
Video Game Questionnaire	–	√		
**Cognitive Battery Module**				
Wechsler Intelligence Scale for Children-IV-Chinese Version (WISC-IV)	6–16	√	√	√
Attention Network Test (ANT)	–	√	√	√
Task-Switch Paradigm	–	√	√	√
Digit N-back Working Memory Task	–	√	√	√

The Chinese Version of WISC-IV was used to measure cognitive ability (Baron, 2005, Watkins and Smith, 2013, Wechsler, 2003, Zhang, 2009). The scale consists of 10 core subtests and 4 supplementary subtests, which provides a full-scale Intelligence Quotient (FSIQ) reflecting the general cognitive ability and four indices: Verbal Comprehension Index (VCI), Perceptual Reasoning Index (PRI), Working Memory Index (WMI) and Processing Speed Index (PSI). Three E-Prime computer tasks, including the Attention Network Test, Task-Switch Paradigm and Digit N-back Task, were used to assess children’s alerting, orienting, executive attention, cognitive flexibility and working memory (Fan et al., 2002, Schuch and Koch, 2003, Zhu et al., 2006).

### 2.4 Imaging measures and analyses

Imaging data at CCNP-SWU were collected across all waves on a Siemens Trio 3 T scanner at Southwest University, while imaging data at CCNP-CAS were collected on a GE Discovery MR750 3 T scanner at the CAS Institute of Psychology. Two resting-state functional MRI (rfMRI) scans were acquired and separated by a T1-weighted MRI scan. The main acquisition parameters for T1-weighted MRI and rfMRI are listed in Supplementary Table S2. A T2-weighted MRI scan was also performed to evaluate brain lesions and improve cross-registration. All the Child Questionnaires and Cognitive Battery Tests were completed after the imaging scans, in order to avoid the influence of physiological fatigue and affective state. Structural MRI images were first anonymized to remove all the personal information from the raw MRI data. We obscured the facial information by using the ***face-masking*** tool (Milchenko and Marcus, 2013) customized with the Chinese pediatric templates developed by our lab (Dong et al., 2020). The anonymized images were then denoised by spatially adaptive non-local means and corrected for intensity normalization in the Connectome Computation System (Xu et al., 2015). To extract individual brains, we trained a deep learning method (Wang et al., 2021) using a small set of semi-automatically extracted brains in the CCNP-SWU samples, and then applied to all the devCCNP samples. The preprocessed brain volumes were all in the native space and fed into FreeSurfer (version 6.0) pipeline (Fischl, 2012) to obtain general morphological measurements of different brain tissues from *aseg.stats* and *lh/rh.aparc.stats* including estimated intracranial volume (eTIV), total gray matter volume (GMV), subcortical gray matter volume (sGMV), cerebral white matter volume (cWMV), mean cortical thickness (CT) and white matter surface area (SA).

It is important to consider MRI instrument and data processing factors when designing multi-site longitudinal studies. We implemented the same ALD for multiple subprojects (i.e., devCCNP-SWU and devCCNP-CAS). Different age groups (cohorts) were tested on same scanners for same subprojects while not on the same scanners for different subprojects. To reduce variability or bias introduced by different scanning platforms, participants from different subprojects would be tested with the same field strength and acquisition parameters. Data and its analytic protocol are harmonized and optimized across different scanners or subprojects for achieving highly reliable multimodal MRI measurements. To ensure growth charts minimally affected by scanning platform, we applied the longitudinal ***ComBat*** method (Beer et al., 2020) to harmonize the extracted morphological measurements prior to the growth curve modelling. Generalized Additive Mixed Model (GAMM) was used to model the brain morphological growth for each sex with the harmonized samples from both devCCNP-SWU and devCCNP-CAS (van Duijvenvoorde et al., 2019). The GAMMs took participant as a random intercept by ***mgcv*** package in **R** 4.1.

## 3 Results

### 3.1 Recruitments and samples

In devCCNP-SWU, both children and their parents/guardians were approached through lectures that explained this large-scale longitudinal cohort and science popularization activities regarding brain development in primary and middle/high schools for students in grades 1–11 (i.e., grade 2 in senior high school). By contrast, devCCNP-CAS comprises community samples recruited via the public uses social media ***wechat*** and science popularization activities. The exclusion criteria included the following: (a) children’s birth weight less than 1.5 kg or greater than 4.2 kg; (b) children with a gestational or perinatal history of pathologies or risk factors; (c) children’s height, weight, or head circumference less than the third percentile of the growth curve of Chinese children and adolescents; (d) children with a history of head injury; (e) children or one of their family members diagnosed with a neuropsychiatric disorder, such as schizophrenia, ASD, ADHD, bipolar disorder, alcohol or other drug use disorder; (f) Wechsler Intelligence Scale for Children-IV (WISC-IV) full-scale Intelligence Quotient (FSIQ) standard score lower than 80. Both children and their parents/guardians volunteered to participate in this study and signed the informed consent before participation.

The devCCNP-SWU recruitment took place between March and December in 2013. A total of 198 children volunteered to take part in this study at baseline, of which 6 were excluded due to brain cyst (*n* = 2), depression disorder (*n* = 1), claustrophobia (*n* = 1), or WISC-IV FSIQ standard score below 80 (*n* = 2). Therefore, 192 children were eligible for further participation, which formed the sample for the baseline assessment of CCNP-SWU. Because of attrition, of the 192 original samples at baseline (Wave 1), 152 participated in the 15-month follow-up (Wave 2), representing a 79.17% re-participation rate from Wave 1 to Wave 2. Seven children were further included at Wave 2; of the 159 children participating at Wave 2, 106 retained in the 30-month follow-up (Wave 3), representing a 66.67% re-participation rate from Wave 2 to Wave 3. Also, 2 children were further included at Wave 3. Eventually, devCCNP-SWU sample consisted of 201 children who participated at least one wave, of which 100 (49.75%) participated in all three waves, 58 (28.86%) participated in two waves, and 43 (21.39%) participated in only one wave.

Data collection of devCCNP-SWU for Wave 1 was conducted from December 2013 to July 2014; data collection for Wave 2 was conducted from April to August in 2015; data collection for Wave 3 was conducted from September 2016 to January 2017. The mean time lag between Wave 1 and Wave 2 was 1.27 years (*SD* = 0.06; range = 1.16–1.50 years), and between Wave 2 and Wave 3 was 1.37 years (*SD* = 0.07; range = 1.22–1.56 years). Attrition analyses were conducted to compare the devCCNP-SWU families who participated in all three measurement waves (*n* = 100) with these who participated in Wave 1 and then dropped out at either Wave 2 or Wave 3 (*n* = 92) on sociodemographic characteristics (see Supplementary Table S1). The attrition analyses indicated that the retained families did not differ from the drop-out families in the distribution of child sex, family size, urban areas or rural areas, and parental education status. However, children who attended measurement 3 times were younger than those who participated in Wave 1 and subsequently dropped out. We also conducted attrition analyses on main psychological outcomes (i.e., problem total scores, personality, social anxiety, depression, and WISC-IV scores) and brain morphological indexes. The results showed that there were no significant differences in the main psychological and brain morphological measures between retained families and drop-out families after FDR correction (FDR corrected *ps* > 0.075).

**Fig. 3:**
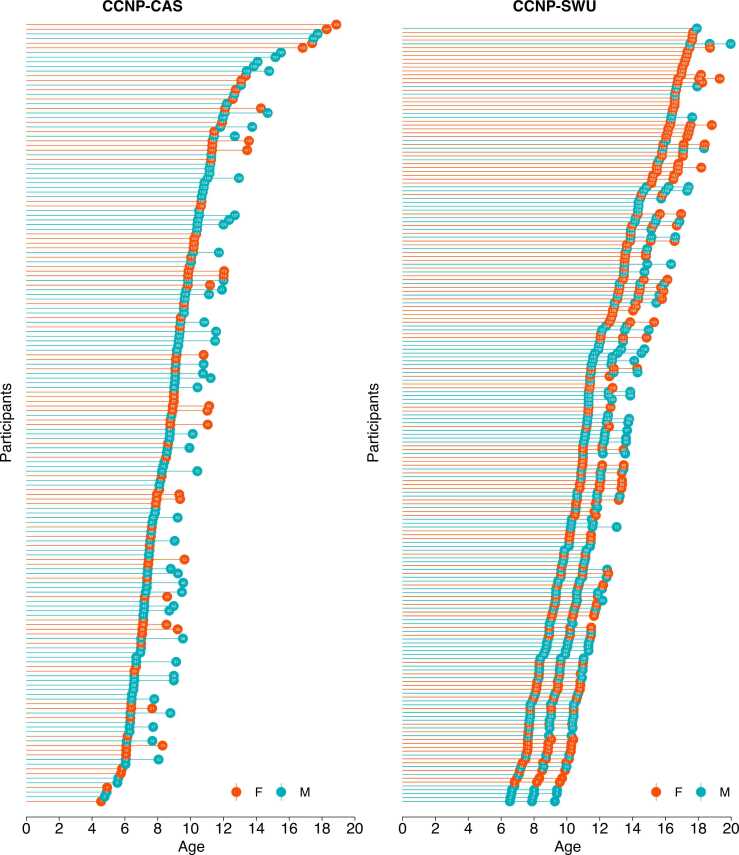
Age and sex distributions of devCCNP-SWU and devCCNP-CAS samples.

The devCCNP-CAS has been recruiting participants since July 2018 and has enrolled 338 children thus far. One child was excluded due to WISC-IV FSIQ standard score below 80. The baseline data have been obtained from 168 eligible children since July 2018, of which 57 have already completed data collection at Wave 2 since September 2020. The rest of the children who have been enrolled but not assessed would be invited to complete the assessment as soon as possible. Fig. 3 presents age and sex distributions of devCCNP-SWU and devCCNP-CAS samples. Sociodemographic characteristics for devCCNP-SWU and devCCNP-CAS samples are summarized in Table 3. Pearson’s Chi-squared tests were used to compare sample characteristics between devCCNP-SWU and devCCNP-CAS samples. The results indicated that children in devCCNP-CAS were more likely to live in urban areas and had highly educated mothers and fathers than children in devCCNP-SWU.

**Table 3:** Sociodemographic characteristics for devCCNP-SWU and devCCNP-CAS samples.

Domain		CCNP-SWU *n* = 201	CCNP-CAS *n* = 168	*χ*^*2*^	*p*
**Child characteristics**					
Child gender					
	Boys	96	97	3.65	0.056
	Girls	105	71		
Only child or not					
	Only child	140	102	1.64	0.201
	Non-only child	54	53		
**Family characteristics**					
Areas					
	Urban areas	146	151	24.50	<0.001
	Rural areas	42	6		
Maternal education					
	Middle school education or less	81	3	179.34	<0.001
	High school education	60	5		
	Associate or bachelor’s degree	51	86		
	Master’s or doctorate degree	3	62		
Paternal education					
	Middle school education or less	66	1	155.81	<0.001
	High school education	55	7		
	Associate or bachelor’s degree	68	77		
	Master’s or doctorate degree	5	71		

**Fig. 4:**
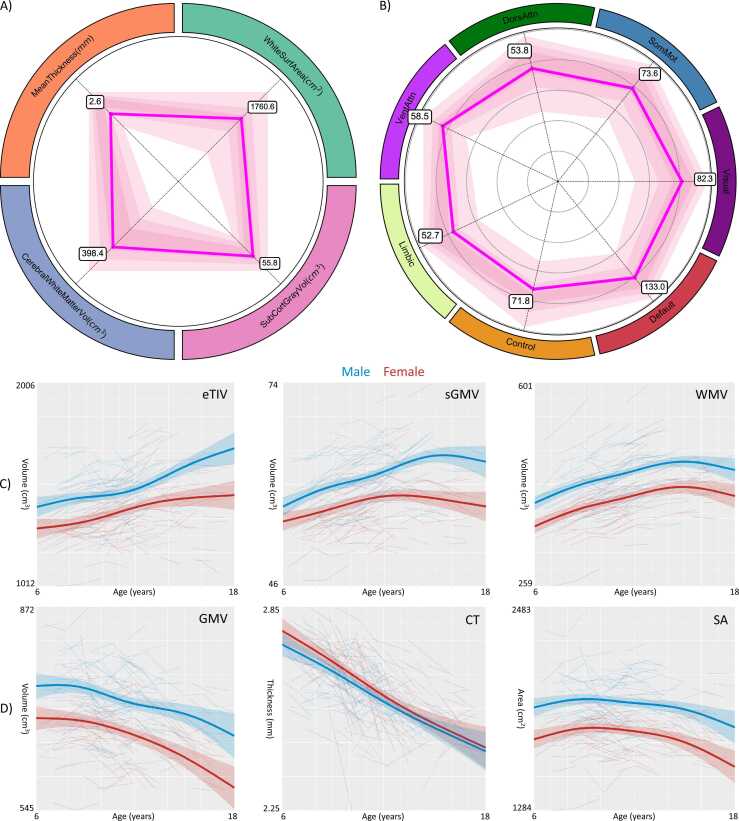
Brain morphological growth curves in schoolchildren. Individual scores are plotted onto the normative growth charts of the global tissue morphological measurements (A) and volumetric measures of the seven common brain networks (see Yeo et al., 2011 for more details on the networks) (B). Growth curves are generated with their confidence intervals for the estimated intracranial volume (eTIV), subcortical gray matter volume (sGMV), cerebral white matter volume (WMV) (C), and total gray matter volume (GMV), mean cortical thickness (CT) and white matter surface area (SA) (D) males and females, respectively. These curves base the growth charts for the individual assessments.

After assessment at each wave, every family received a personal development report for participation. The development report provided feedback on five aspects of children’s development, including physiological characteristics (i.e., height, weight, head circumference, blood pressure, and pulse), cognitive ability, social-emotional development (e.g., social anxiety, depression, stress perception and behavioral problems), personality, and brain development. The brain development report included the global tissue morphology (i.e., eTIV, GMV, sGMV, cWMV) and the network-level volumetric features (Yeo et al., 2011). The parents were shown a percentile score for their child (see Fig. 4A and B for a sample report of a girl). We note that this is an objective report of the test results and blanket recommendations instead of interventional strategies that may alter the behaviors of the participants or their parents. In addition, we emphasized that the participant’s performance was always affected by the physical and emotional state while their developmental processes are dynamic and more informative and valid based on the final reports derived from the 5-year following assessments.

### 3.2 Key findings

Main variables of boys and girls across two age groups (younger group less than 12 years old vs. older group greater than 12 years old) at devCCNP-SWU baseline are presented in Table 4. The raw scores of extraversion, neuroticism and psychoticism assessed by Eysenck Personality Questionnaire (EPQ) were used. Independent samples *t*-tests were conducted to compare the means of main variables between boys and girls across two age groups. There were sex differences in body mass index (BMI), CBCL problem total scores, depression, neuroticism, psychoticism, eTIV, GMV, sGMV, cWMV, CT and SA in younger group. As for the older group, there were significant sex differences in height, social anxiety measured by Social Anxiety Scale for Children (SASC), psychoticism, FSIQ, verbal comprehension, processing speed, eTIV, GMV, sGMV, cWMV, and SA. Table 5 presents main variables of boys and girls across two age groups at devCCNP-CAS baseline. There were detectable sex differences in processing speed, eTIV, GMV, sGMV, cWMV, and SA for younger group, and in cWMV and SA for older group.

**Table 4:** Main variables of boys and girls across two age groups at devCCNP-SWU baseline (*N* = 192).

Domain		Younger Group		*p*	Older Group		*p*
		Boys *n* = 64	Girls *n* = 47		Boys *n* = 28	Girls *n* = 53	
**Biophysical Measures**							
Height (m)		1.39 ± 0.11	1.36 ± 0.09	0.157	1.62 ± 0.10	1.57 ± 0.05	0.022
Weight (kg)		37.51 ± 12.61	31.25 ± 7.63	0.002	55.02 ± 12.29	50.60 ± 6.67	0.090
BMI (kg/m^2^)		19.09 ± 4.04	16.73 ± 2.48	<0.001	20.18 ± 3.25	20.57 ± 2.68	0.580
**Questionnaires**							
CBCL Problem Total Scores		25.05 ± 16.26	17.89 ± 12.51	0.016	26.22 ± 20.49	21.06 ± 16.66	0.264
Social Anxiety		4.71 ± 4.24	4.72 ± 3.99	0.989	7.09 ± 3.06	8.83 ± 3.61	0.047
Depression		10.58 ± 6.86	7.98 ± 5.09	0.031	12.06 ± 6.46	13.15 ± 5.18	0.533
EPQ Raw Scores							
	Extraversion (E)	17.27 ± 3.45	16.85 ± 4.01	0.560	14.75 ± 6.57	16.23 ± 4.24	0.288
	Neuroticism (N)	6.17 ± 4.05	4.17 ± 3.06	0.004	8.43 ± 4.58	9.79 ± 5.04	0.236
	Psychoticism (P)	2.83 ± 2.34	1.53 ± 1.37	<0.001	3.04 ± 2.15	2.04 ± 1.63	0.022
**Cognitive Battery Module**							
WISC-IV Scores							
	FSIQ	107.52 ± 10.49	110.11 ± 12.25	0.234	110.04 ± 10.56	117.23 ± 12.48	0.016
	VCI	112.17 ± 14.02	115.79 ± 13.77	0.179	120.00 ± 13.98	131.02 ± 16.94	0.007
	PRI	105.52 ± 10.64	106.96 ± 12.37	0.512	108.00 ± 13.63	107.32 ± 13.73	0.841
	WMI	98.41 ± 10.36	98.11 ± 8.82	0.873	98.08 ± 10.45	101.07 ± 11.92	0.293
	PSI	105.02 ± 13.38	107.49 ± 14.75	0.359	99.69 ± 8.00	108.93 ± 15.92	0.002
**Brain morphological indexes**							
eTIV		1445.49 ± 125.63	1304.57 ± 103.36	<0.001	1492.30 ± 152.87	1367.61 ± 143.53	0.001
GMV		709.35 ± 49.64	648.63 ± 46.71	<0.001	695.99 ± 52.67	633.81 ± 44.78	< 0.001
sGMV		60.28 ± 4.02	55.89 ± 3.72	<0.001	61.89 ± 4.77	57.19 ± 2.99	< 0.001
cWMV		435.40 ± 42.60	388.24 ± 38.46	<0.001	453.56 ± 46.46	422.21 ± 36.61	0.002
CT		2.58 ± 0.08	2.62 ± 0.08	0.008	2.52 ± 0.06	2.50 ± 0.08	0.425
SA		1866.74 ± 141.44	1676.81 ± 124.95	<0.001	1859.98 ± 170.86	1696.18 ± 122.09	< 0.001
eTIV (combat)		1459.06 ± 127.99	1315.97 ± 104.26	<0.001	1506.96 ± 155.30	1385.56 ± 146.05	0.002
GMV (combat)		735.64 ± 51.14	674.49 ± 47.77	<0.001	721.29 ± 54.05	659.75 ± 46.95	< 0.001
sGMV (combat)		60.25 ± 4.10	55.89 ± 3.78	<0.001	62.05 ± 4.86	57.42 ± 3.07	< 0.001
cWMV (combat)		430.85 ± 43.04	383.27 ± 38.86	<0.001	450.98 ± 47.09	420.10 ± 37.37	0.003
CT (combat)		2.64 ± 0.08	2.68 ± 0.08	0.010	2.57 ± 0.07	2.55 ± 0.09	0.403
SA (combat)		1897.71 ± 144.22	1705.76 ± 127.61	<0.001	1891.63 ± 173.43	1729.55 ± 124.82	< 0.001

**Table 5:** Main variables of boys and girls across two age groups at devCCNP-CAS baseline (*N* = 168).

Domain		Younger Group		*p*	Older Group		*p*
		Boys *n* = 86	Girls *n* = 62		Boys *n* = 11	Girls *n* = 9	
**Biophysical Measures**							
Height (m)		1.36 ± 0.13	1.34 ± 0.13	0.593	1.70 ± 0.13	1.63 ± 0.05	0.128
Weight (kg)		31.74 ± 9.51	30.06 ± 10.96	0.322	59.46 ± 20.45	58.18 ± 10.47	0.866
BMI (kg/m^2^)		16.96 ± 2.95	16.17 ± 2.95	0.112	20.01 ± 4.01	21.89 ± 3.30	0.275
**Questionnaires**							
CBCL Problem Total Scores		24.28 ± 17.77	18.95 ± 18.48	0.088	17.55 ± 9.63	21.71 ± 24.18	0.676
Social Anxiety		3.96 ± 2.95	5.31 ± 5.18	0.106	6.22 ± 5.59	6.14 ± 4.91	0.977
Depression		9.72 ± 6.46	9.19 ± 7.49	0.693	11.27 ± 7.56	10.86 ± 6.94	0.908
EPQ Raw Scores							
	Extraversion (E)	18.73 ± 3.85	17.54 ± 3.73	0.152	18.29 ± 3.30	19.20 ± 3.35	0.648
	Neuroticism (N)	5.55 ± 5.97	5.63 ± 5.57	0.944	7.29 ± 7.09	7.60 ± 5.90	0.937
	Psychoticism (P)	3.34 ± 3.32	2.24 ± 2.54	0.092	4.43 ± 4.12	4.60 ± 5.94	0.954
**Cognitive Battery Module**							
WISC-IV Scores							
	FSIQ	116.54 ± 13.15	119.51 ± 14.71	0.224	116.56 ± 12.95	126.25 ± 17.46	0.284
	VCI	119.35 ± 14.80	116.77 ± 17.98	0.374	124.67 ± 21.07	133.00 ± 22.58	0.532
	PRI	116.12 ± 14.62	117.28 ± 14.70	0.656	111.67 ± 14.14	123.75 ± 6.65	0.137
	WMI	109.53 ± 13.07	113.28 ± 12.53	0.105	104.11 ± 10.46	112.25 ± 21.27	0.362
	PSI	100.34 ± 14.24	109.00 ± 15.19	0.001	104.44 ± 11.63	104.00 ± 8.83	0.947
**Brain morphological indexes**							
eTIV		1491.81 ± 120.69	1400.30 ± 99.01	<0.001	1598.32 ± 196.02	1433.08 ± 92.25	0.073
GMV		775.75 ± 57.19	724.93 ± 41.46	<0.001	769.63 ± 72.90	704.86 ± 43.09	0.067
sGMV		59.90 ± 4.35	57.66 ± 3.67	0.005	63.54 ± 5.52	59.75 ± 4.43	0.170
cWMV		433.28 ± 43.06	393.43 ± 32.76	<0.001	464.76 ± 53.15	409.92 ± 26.11	0.033
CT		2.72 ± 0.07	2.73 ± 0.07	0.397	2.67 ± 0.08	2.70 ± 0.08	0.346
SA		1944.62 ± 154.98	1798.45 ± 117.79	<0.001	1939.04 ± 195.60	1734.28 ± 82.25	0.029
eTIV (combat)		1446.52 ± 128.26	1356.83 ± 103.59	<0.001	1558.27 ± 209.80	1393.66 ± 96.86	0.092
GMV (combat)		719.33 ± 61.08	668.93 ± 43.87	<0.001	712.85 ± 77.00	649.47 ± 47.35	0.089
sGMV (combat)		59.36 ± 4.47	57.20 ± 3.75	0.008	63.15 ± 5.56	59.40 ± 4.52	0.179
cWMV (combat)		436.86 ± 43.92	397.21 ± 33.66	<0.001	469.55 ± 54.47	414.83 ± 26.82	0.037
CT (combat)		2.60 ± 0.09	2.61 ± 0.09	0.497	2.54 ± 0.10	2.58 ± 0.10	0.364
SA (combat)		1875.25 ± 160.63	1728.36 ± 121.79	<0.001	1870.64 ± 201.00	1666.34 ± 83.80	0.033

During the outbreak of Coronavirus Disease-2019 (COVID-19), we did not collect devCCNP data from January to August in 2020. Data collection has returned to normal since September 2020. However, we designed questionnaires to collect data on children’s learning and daily life during COVID-19 pandemic (see Table 6). A total of 66 children (mean age = 11.38 years, *SD* = 3.33; 32 boys, 34 girls) from devCCNP-SWU (*n* = 11) and devCCNP-CAS (*n* = 55) completed the questionnaires online from March to October in 2020. About three in five children (59.09%) felt a bit nervous or panic when COVID-19 spread rapidly. The COVID-19 pandemic exerted slight impact on daily routine for more than 60% of the households (62.12%), and exerted moderate or strong impact on daily routine for nearly 30% of the households (28.79%). As illustrated in Table 6, more than half of the children reported that their learning efficiency, learning consciousness and exercise time were decreased. Meanwhile, screen time was dramatically increased for more than 60% of the children. Children’s emotional states during COVID-19 pandemic were also measured by four scales: Depression Anxiety Stress Scale (DASS; Lovibond and Lovibond, 1995), Children’s Depression Inventory (CDI; Kovacs, 1992), Depression Self-rating Scale for Children (DSRSC; Birleson, 1981), and Childhood Anxiety Sensitivity Index (CASI; Silverman et al., 1999). The descriptive statistics were presented in Supplementary Table S3.

**Table 6:** The impact of COVID-19 on children’s learning and daily life (*N* = 66).

	Decreased dramatically	Decreased slightly	Remained about the same	Increased slightly	Increased dramatically
Children’s learning time	8 (12.12%)	21 (31.82%)	19 (28.79%)	12 (18.18%)	6 (9.09%)
Children’s learning stress	10 (15.15%)	11 (16.67%)	31 (46.97%)	10 (15.15%)	4 (6.06%)
Children’s learning efficiency	10 (15.15%)	28 (42.42%)	14 (21.21%)	8 (12.12%)	6 (9.09%)
Children’s learning consciousness	12 (18.18%)	22 (33.33%)	20 (30.30%)	7 (10.61%)	5 (7.58%)
Children’s exercise time	26 (39.39%)	14 (21.21%)	13 (19.70%)	5 (7.58%)	8 (12.12%)
Children’s fun time	3 (4.55%)	5 (7.58%)	26 (39.39%)	17 (25.76%)	15 (22.73%)
Children’s screen time	3 (4.55%)	0 (0%)	8 (12.12%)	14 (21.21%)	41 (62.12%)
Parental involvement in children’s learning	4 (6.06%)	2 (3.03%)	33 (50.00%)	13 (19.70%)	14 (21.21%)
Parent-child interaction time	5 (7.58%)	7 (10.61%)	33 (50.00%)	12 (18.18%)	9 (13.64%)
Parental conflicts	7 (10.61%)	6 (9.09%)	44 (66.67%)	8 (12.12%)	1 (1.52%)
Parent-child conflicts	9 (13.64%)	6 (9.09%)	37 (56.06%)	12 (18.18%)	2 (3.03%)

Growth curves are generated with both site-harmonized and raw data. While the overall shapes the growth curves are similar, the longitudinal *ComBat* improves the growth curve modeling, showing more powered longitudinal changes. In the main text, we visualized the growth curves using the *ComBat* derived data in Fig. 4 (see Supplementary Figure 1 for those using the raw data). Specifically, eTIV, sGMV and WMV exhibited a growth pattern with increasing volumes when growing up (Fig. 4C). In contrast, CT was growing as a linearly thinning process with development while the growth curves of GMV and SA demonstrated somehow nonlinearity, indicating an inverted-U growing shape (Fig. 4D).

### 3.3 Publications

A previous review was published in Chinese by our group, which comprehensively described the devCCNP-SWU protocol for experimental design, sample selection and data collection (Yang et al., 2017). Using the devCCNP-SWU baseline brain imaging data from 84 participants, we previously reported that children exhibited similar region-specific asymmetry of dorsal anterior cingulate cortex (dACC) as in adults, and further revealed that dACC functional connectivity with default, frontoparietal and visual networks showed a region-specific asymmetry (Wang et al., 2015). This baseline data has been released as part of the Consortium for Reliability and Reproducibility (CoRR; Zuo et al., 2014), the IPCAS 7 site (http://dx.doi.org/10.15387/fcp_indi.corr.ipcas7), which has been listed as one of the existing, ongoing large-scale developmental dataset (Rosenberg et al., 2018). Head motion data during mock-scanning from devCCNP-CAS are recently demonstrated with frequency-specific evidence to support motion as a developmental trait across children and adolescents by the development of a neuroinformatic tool, namely DREAM (Gong et al., 2021).

The full set of devCCNP data is increasingly appreciated by collaborative studies on school-aged children and adolescents. It supports the building of age-specific cranio-cortical correspondences for school-aged children and adolescents (Zhang et al., 2021). We recently observed that social anxiety was positively correlated with the GMV in an area of the orbital-frontal cortex, and its functional connectivity with the amygdala (Mao et al., 2020). Using the longitudinal data, we also charted the growth curves of human amygdala across school ages (Zhou et al., 2021). A standardized protocol on charting brain development during school age has been developed to generate the corresponding brain templates and model growth charts, revealing the differences in brain morphological growth between Chinese and American population, in particularly around puberty (Dong et al., 2020). Using resting-state fMRI data from devCCNP-SWU, we revealed age-dependent changes in the macroscale organization of cortex. The findings suggest that the scheduled maturation of functional connectivity gradient shifts may be critically important for understanding how cognitive and behavioral capabilities are refined across development, marking puberty-related changes (Dong et al., 2021). As part of an international consortium for human lifespan brain chart recently initiated (Bethlehem et al., 2021), CCNP contributes to the largest world-wide MRI samples (N > 120,000) for building the normative brain charts for the human lifespan (0–100 years).

## 4 Discussion

CCNP is a cohort to combine longitudinal brain imaging, biophysical, social, behavioral and cognitive measures across nearly the full lifespan, enhancing the culture diversity among the existing accelerated longitudinal datasets (Table 1). The pilot CCNP demonstrated that the accelerated longitudinal and freely available data on high-resolution structural and functional images beginning in childhood and extending into early adulthood with the same imaging protocols have been practical to acquire. Such data are invaluable for the construction of normative growth charts to describe how brain morphological metrics and functional connectivity change as a function of child age across sexs (Zuo et al., 2017, Dong et al., 2020, Holla et al., 2020a, Holla et al., 2020; Qiu, 2020). In addition, longitudinal psycho-behavioral data will enable the scientific community to investigate how age-related changes in brain structure and function are associated with age-related changes in cognitive function and behavioral performances. By harnessing big data, the field of developmental cognitive neuroscience is rapidly transitioning into a population science, namely developmental population cognitive neuroscience (Zuo et al., 2018). This raises lots of challenges and opportunities (Becht and Mills, 2020, Fair et al., 2021, Marek et al., 2020, Mills and Tamnes, 2014), such as the how to combine the existing data resources and delineating the individual differences in brain and mind development. One promising solution would be to plan more comprehensive and sophisticated large-scale national-level cohort based upon the rich experiences from the existing cohort. ABCD (Garavan et al., 2018) and Lifebrain (Walhovd et al., 2018) are two national projects using longitudinal designs in the United States and Europe, respectively. In China, the National Brain Project (Poo et al., 2016) has initiated a large longitudinal cohort on school-aged brain and mind development. The devCCNP has shared its experiences in building the national cohort, and in future, will distribute its initial practice into other 48 sites across the country for a final national representative sample including 50 sites (i.e., 38,000 participants, 114,000 samples). This will build normative charts for translating the basic research on individual differences in brain and mind development into educational and clinical conditions across the lifespan (Bethlehem et al., 2021). We also hope that the final CCNP will offer a unique resource for examining cultural or ethnic effects on development of brain and mind (Fan et al., 2015; Qiu, 2020).

This study has several limitations. First, we are aware of the inevitable sample attrition across three waves in CCNP-SWU. Although attrition analyses suggested that child sex and family background factors did not contribute to the attrition, children who completed all three waves were younger than those who participated in Wave 1 and then dropped out of the study at either Wave 2 or Wave 3, which may introduce bias, especially when constructing growth curves and describing age-related changes. Second, CCNP focuses exclusively on Chinese Han population, and therefore the findings may not be generalizable to other ethnic groups. There is a physiological variation between different ethnic groups, and cognitive neuroscience research on ethnic minority groups in China is also an important direction for future studies. Finally, the sample is limited to a few regions, which may limit its ability to represent Chinese general population. Due to the complexity and high cost of MRI brain scanning, it is difficult to carry out large-scale and coordinated nationwide research, which highlights the need for conducting collaborative research and promoting MRI data sharing.

Baseline CCNP-SWU data on brain imaging are available to researchers via the CoRR, which is committed to open science by aggregating and sharing MRI data from multiple sources to establish test-retest reliability and reproducibility in functional connectomics (Zuo et al., 2014). The rest of the data will be publicly shared via the National Science Data Bank and fully available to the research community when acquisition is completed for the pilot CCNP. At this stage, data are only available to researchers and collaborators of CCNP. More information about CCNP can be found at: http://deepneuro.bnu.edu.cn/?p=163 or https://github.com/zuoxinian/CCNP. Requests for further information and collaboration are encouraged and considered by principal investigator Xi-Nian Zuo [xinian.zuo@bnu.edu.cn].

## The chinese color nest consortium

The Chinese Color Nest Consortium members are at http://deepneuro.bnu.edu.cn/?p=163.

## Funding

This work was supported by the Start-up Funds for Leading Talents at Beijing Normal University, the Key-Area Research and Development Program of Guangdong Province (2019B030335001), the National Basic Science Data Center “Chinese Data-sharing Warehouse for In-vivo Imaging Brain” (NBSDC-DB-15), the 10.13039/501100009592Beijing Municipal Science and Technology Commission (Z161100002616023, Z181100001518003), the Major Project of National Social Science Foundation of China (20&ZD296), the CAS-NWO Programme (153111KYSB20160020), the Guangxi BaGui Scholarship (201621) and National Basic Research (973) Program (2015CB351702), 10.13039/501100001809National Natural Science Foundation of China (Major Fund for International Collaboration: 81220108014), the Chinese Academy of Sciences Key Research Program (CAS: KSZD-EW-TZ-002) and the National Basic Research Program (973 Program: 2015CB351702).

## Declaration of Competing Interest

The authors declare that they have no known competing financial interests or personal relationships that could have appeared to influence the work reported in this paper.

## Data Availability

Baseline CCNP-SWU data on brain imaging are available to researchers via the Consortium for Reliability and Reproducibility (http://dx.doi.org/10.15387/fcp_indi.corr.ipcas7), which is committed to open science by aggregating and sharing MRI data from multiple sources to establish test-retest reliability and reproducibility in functional connectomics. The rest of the data will be publicly shared and fully available to the research community when acquisition is completed. At this stage, data are only available to researchers and collaborators of CCNP. More information about CCNP can be found at https://github.com/zuoxinian/CCNP. Requests for further information and collaboration are encouraged and considered by the principal investigator (PI) Xi-Nian Zuo [xinian.zuo@bnu.edu.cn or zuoxn@psych.ac.cn].
