# Supplementary figures and images for: Chinese Color Nest Project : An accelerated longitudinal brain-mind cohort
Source: Dev Cogn Neurosci. 2021 Oct 11;52:101020. doi: 10.1016/j.dcn.2021.101020 (PMC8517840; doi:10.1016/j.dcn.2021.101020)

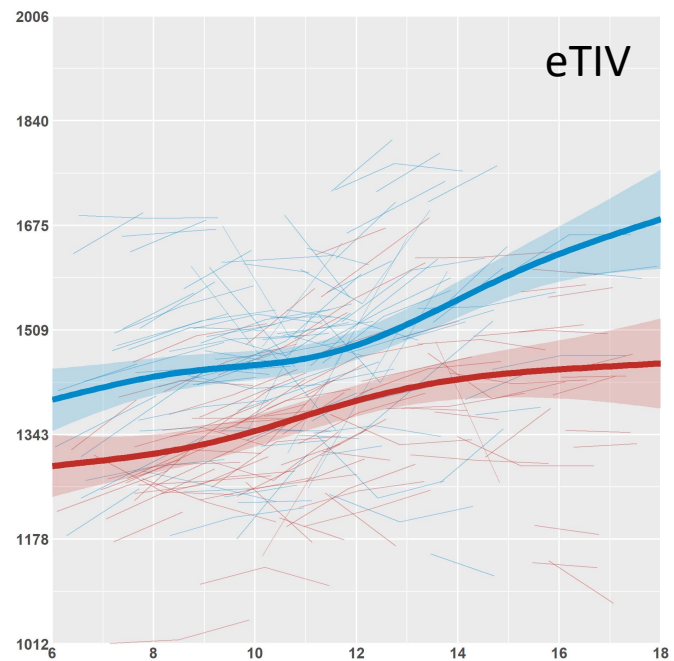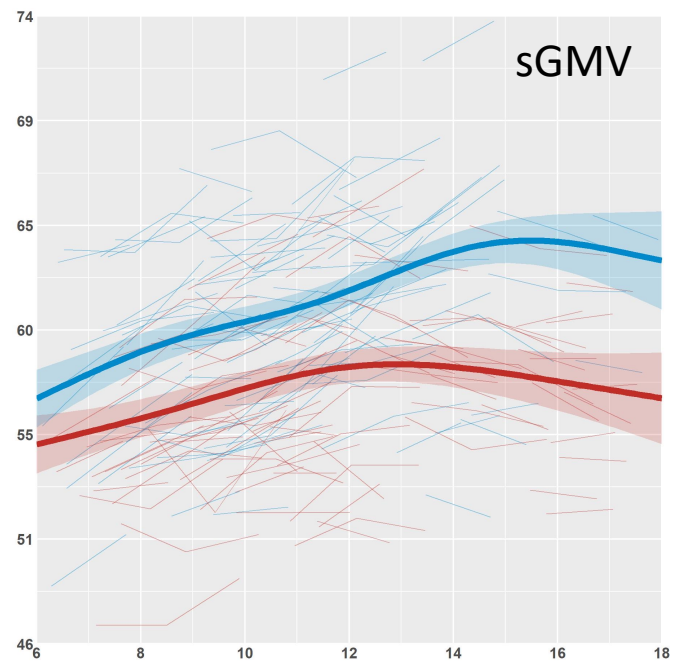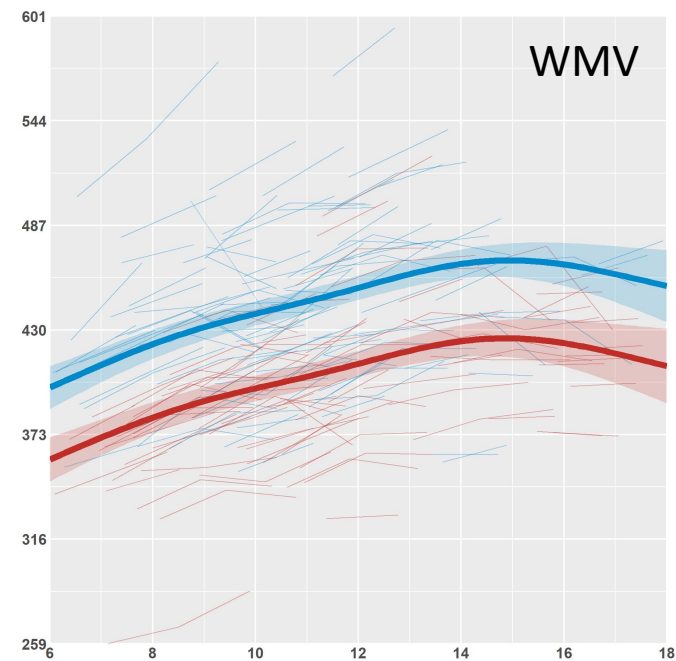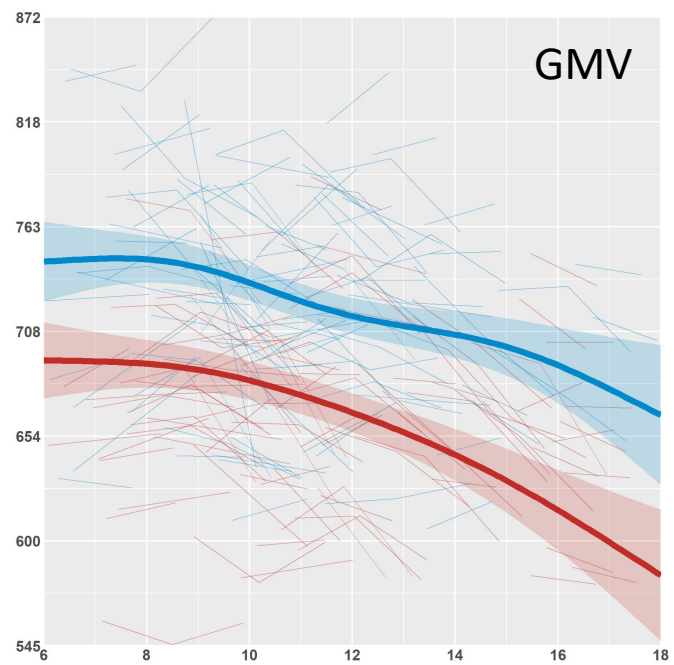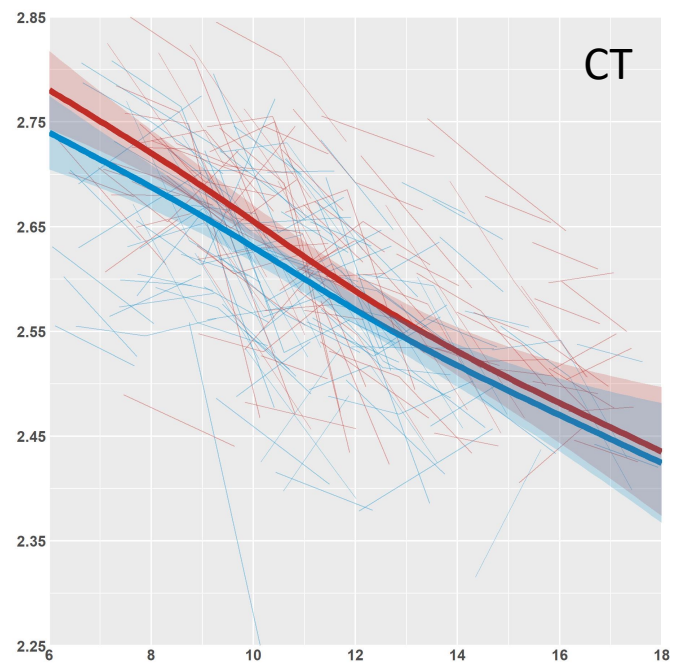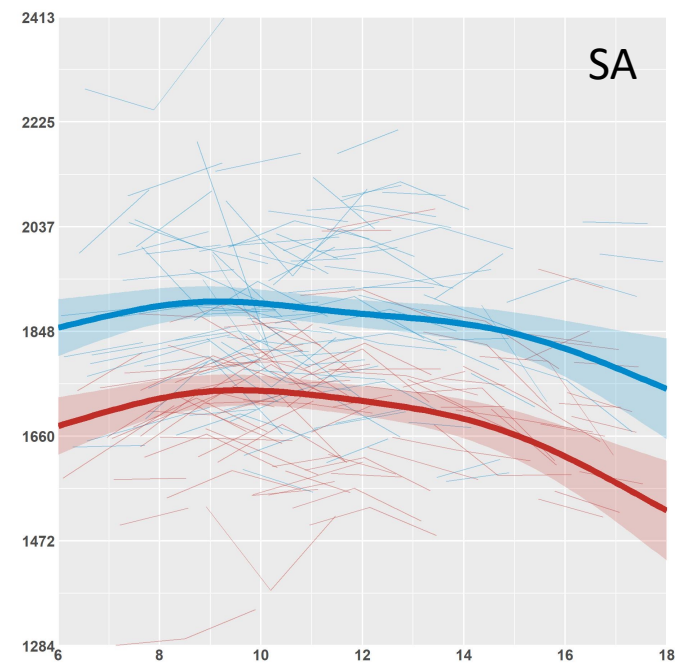

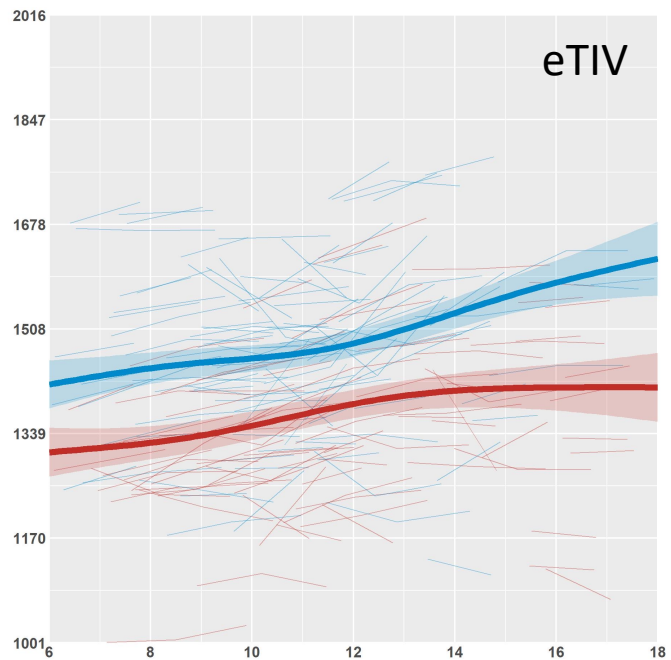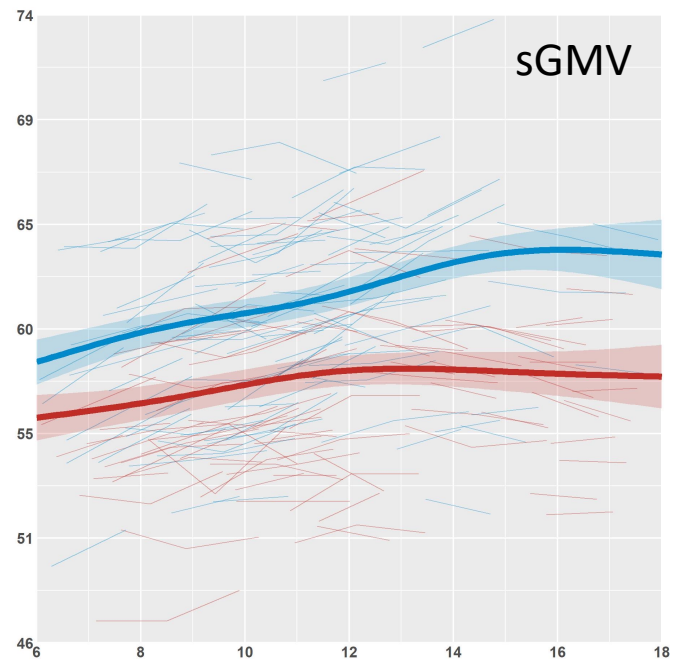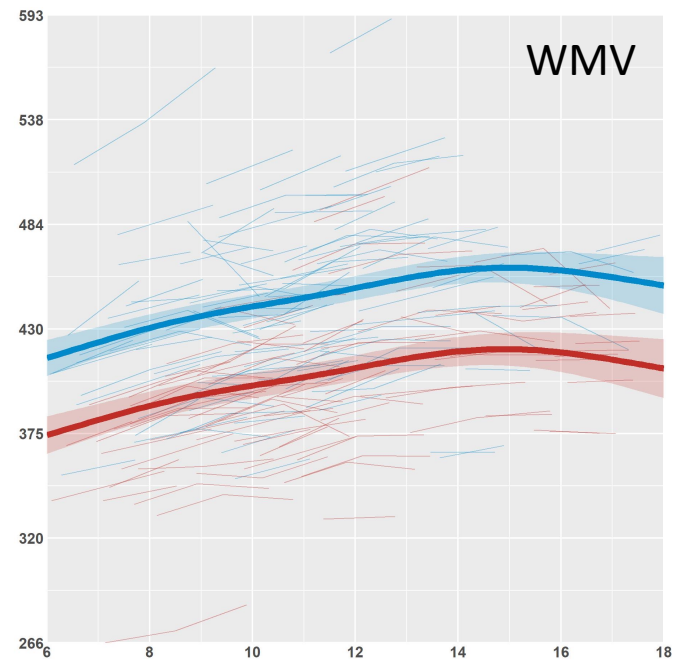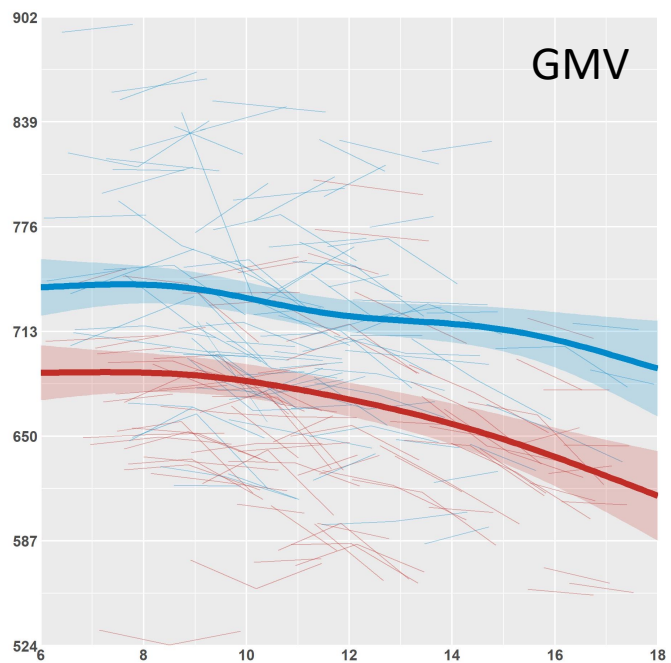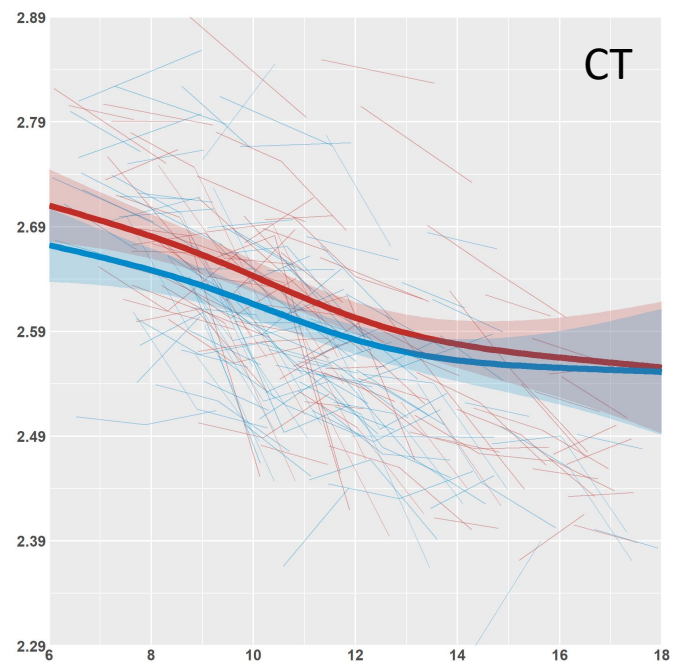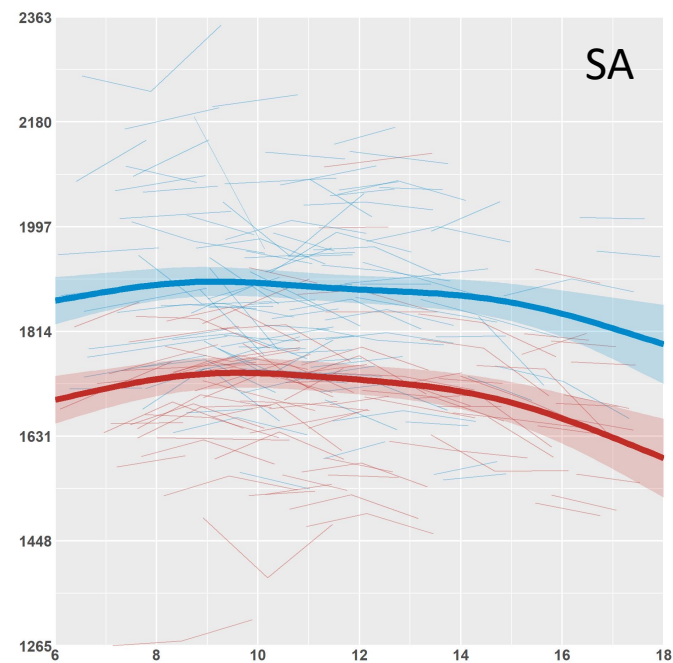

Supplement: Supplementary Table S1 — Supplementary material [file mmc1.pdf]
